# STDR-Net: A Spatio-Temporal Dependency Refinement Network for Motor Imagery EEG Decoding

**DOI:** 10.3390/brainsci16101051

**Published:** 2026-09-29

**Authors:** Wei Zhao, Jianing Dai, Yitong Pan, Shixiao Xiao, Tongle Xie, Yao Li, Yongkang Yu

**Affiliations:** Chengyi College, Jimei University, Xiamen 361021, China

**Keywords:** motor imagery EEG, brain–computer interface, dependency refinement, Transformer, bidirectional LSTM, convolutional neural network

## Abstract

**Background/Objectives:** Motor imagery electroencephalography (MI-EEG) contains weak, non-stationary patterns that evolve across channels and time. Effective decoding requires local sensorimotor structure, trial-wide context, and ordered temporal evolution to be represented without discarding information from earlier processing stages. **Methods:** We propose STDR-Net, a Spatio-Temporal Dependency Refinement Network that recalibrates convolutional tokens, models their global interactions, restores the original local representation through a cross-stage residual bridge, and refines the fused sequence with a bidirectional long short-term memory network. STDR-Net was evaluated on BCI Competition IV-2a, BCI Competition IV-2b, and the PhysioNet EEG Motor Movement/Imagery dataset against nine reproduced baselines. The PhysioNet experiment used a pooled mixed-participant trial-level split rather than an unseen-participant evaluation. **Results:** STDR-Net obtained accuracies of 82.69%, 86.99%, and 59.76% and Cohen’s kappa values of 0.7692, 0.7398, and 0.46, respectively. It achieved the highest observed mean accuracy on IV-2a and competitive performance on IV-2b and PhysioNet. Bridge-specific, ordering, module, and augmentation ablations supported the proposed pathway on IV-2a, whereas the differences on IV-2b were small and not significant after multiplicity correction. **Conclusions:** The results support information-preserving dependency refinement as a competitive design for MI-EEG decoding, while also showing that its benefit depends on the dataset and evaluation setting.

## 1. Introduction

Brain–computer interfaces (BCIs) translate neural activity into control commands and support applications in neurorehabilitation, assistive robotics, and human–machine interaction [1]. Motor imagery (MI) electroencephalography (EEG) is a widely studied non-invasive BCI paradigm because it requires users only to imagine a movement and can be recorded with portable equipment. Imagined movements modulate sensorimotor rhythms through event-related desynchronisation and synchronisation, providing a physiological basis for decoding movement intention [2]. MI-related patterns are nevertheless weak, non-stationary, and highly subject-dependent, which makes reliable single-trial decoding difficult [3,4].

An MI trial is a temporally evolving sensorimotor process. Discriminative information is distributed across electrodes, frequency-selective responses, and successive temporal phases. An effective decoder should therefore preserve local spatio-temporal structure while relating temporally distant events and retaining their order. Aggressive global aggregation can suppress subtle local patterns, whereas purely local processing can miss dependencies beyond a limited receptive field.

Conventional MI-EEG pipelines separate feature extraction from classification. Common spatial pattern (CSP) maximises class-dependent variance differences, and filter-bank CSP extends this principle across multiple frequency bands [5,6]. Deep convolutional networks learn task-relevant filters directly from raw or minimally processed EEG. ShallowConvNet and DeepConvNet introduced end-to-end temporal and spatial convolution, while EEGNet provided a compact architecture based on depthwise and separable convolutions [7,8]. Later methods broadened local representation learning through mixed-scale, multi-branch, and attention-based processing. ADFCNN, for example, combines attention with dual-scale convolutional fusion [9]. Convolution remains effective for local structure, but its finite receptive field does not directly model all trial-wide interactions.

Recurrent networks explicitly model ordered EEG dynamics [10]. Temporal-convolutional approaches enlarge the effective receptive field through stacked or dilated operations [11]. Transformers complement these methods by allowing each temporal token to interact with all other tokens. EEG Conformer combines convolutional tokenisation with Transformer encoding [12], while MSCFormer uses multi-scale convolutional features with Transformer modelling [13]. CLTNet further illustrates the recent use of convolutional, recurrent, and Transformer components in a hybrid MI decoder [14]. These models show the value of local, global, and ordered processing, but the way information is transferred between stages remains important: global mixing may alter local evidence that a later temporal refiner could still be needed.

We therefore propose STDR-Net, an information-preserving dependency-refinement pathway. A convolutional front-end converts each trial into compact temporal tokens, and a squeeze-and-excitation (SE) block recalibrates their feature channels. A Transformer encoder then captures trial-wide interactions. A cross-stage residual bridge adds the original convolutional tokens to the contextualised sequence before a BiLSTM performs bidirectional temporal refinement.

The main contributions are as follows:

1. We introduce a dependency-refinement pathway in which a cross-stage residual bridge gives the BiLSTM direct access to both the original convolutional representation and the Transformer context.
2. We conduct a unified evaluation against nine reproduced baselines on three public MI-EEG datasets. STDR-Net achieves the highest observed mean accuracy on BCI Competition IV-2a and competitive performance on BCI Competition IV-2b and PhysioNet.
3. We systematically evaluate the proposed design through bridge-specific, ordering, module, and augmentation ablations, hyperparameter-sensitivity analyses, statistical comparisons, and computational-cost profiling.

## 2. Related Work

### 2.1. Conventional and Convolutional MI-EEG Decoding

Conventional MI-EEG pipelines commonly separate feature extraction from classification. CSP and filter-bank CSP remain physiologically interpretable benchmarks, but their performance depends on preprocessing, band selection, and subject-specific parameter choices [5,6]. Temporally constrained sparse group spatial patterns add temporal structure and sparsity [15].

Deep convolutional models learn temporal filters and spatial projections jointly. ShallowConvNet, DeepConvNet, EEGNet, MBEEGNet, and subsequent multi-feature networks have explored compact convolution, multiple scales, and feature-channel attention [7,8,16,17,18,19,20]. ADFCNN uses attention-based dual-scale feature fusion to combine complementary convolutional representations [9]. These methods provide strong local feature extraction but do not directly impose trial-wide pairwise interaction modelling.

### 2.2. Sequential and Temporal-Convolutional Modelling

LSTM- and GRU-based architectures use gated memory to represent ordered EEG dynamics, and MI-specific hybrid CNN–recurrent models combine local feature extraction with state propagation [10,21]. Bidirectional recurrent modelling has also been applied to broader EEG decoding tasks, including epileptic seizure detection [22]. Temporal convolutional networks provide an alternative through causal or dilated convolutions; EEG-TCNet and temporal-convolution fusion models enlarge the effective receptive field while retaining parallel computation [11,23,24]. Physics-informed attention, dynamic convolution, and efficient channel-attention mechanisms further improve the selection of informative responses [25,26,27].

### 2.3. Global Interaction and Hybrid Architectures

Transformers model content-dependent interactions between all compressed tokens. EEG Conformer combines convolutional tokenisation, self-attention, and class-activation visualisation [12]. Multi-scale convolutional Transformers, MSCFormer, TCANet, TFCA-TransNet, and CTNet integrate multi-scale, temporal-attention, time–frequency–spatial, or channel-attention mechanisms [13,28,29,30,31]. CLTNet combines convolutional, recurrent, and Transformer processing in a recent hybrid architecture [14]. Graph-based MI methods model inter-electrode relationships [32,33,34], while attention-based fusion and alignment methods address inter-subject variability [35,36].

STDR-Net uses familiar components but a different interface between them. SE recalibrates the local features before global attention; the Transformer establishes trial-wide context; the residual bridge restores the pre-attention convolutional tokens; and the BiLSTM refines this fused sequence. The bridge and ordering ablations in Section 4.1 test whether this information path contributes beyond the capacity of a generic CNN–Transformer–RNN combination.

## 3. Materials and Methods

### 3.1. Overall Architecture

**Figure 1:**
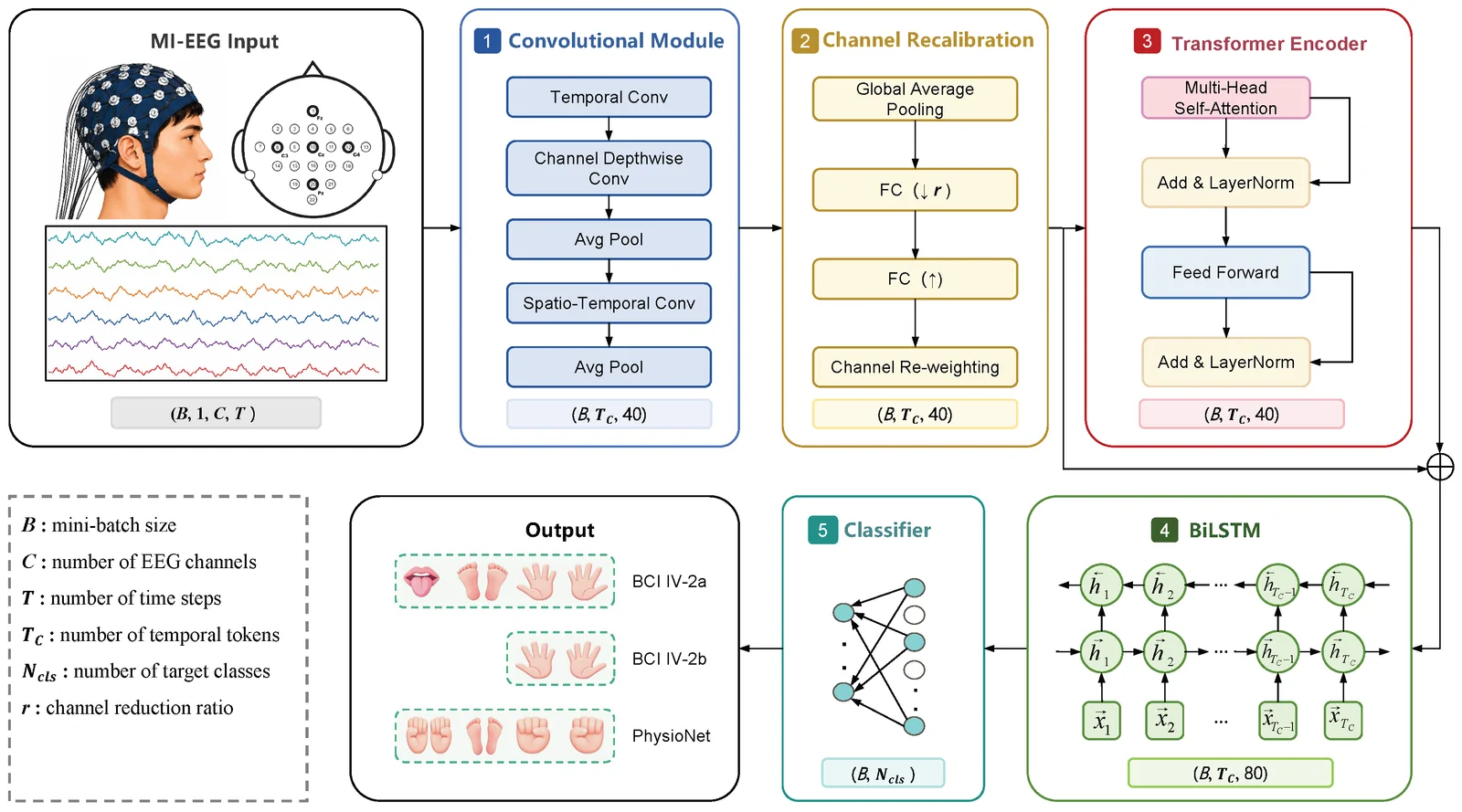
Overall architecture of STDR-Net. The cross-stage residual bridge adds the original convolutional tokens to the Transformer output before BiLSTM refinement.

Figure 1 shows STDR-Net, and Table 1 summarises its layer-wise configuration. Let $X\in R^{B\times 1\times C\times T}$ denote a batch of EEG trials, where *B*, *C*, and *T* are the batch size, number of electrodes, and number of samples. The convolutional front-end extracts local temporal and spatial patterns and compresses the signal into temporal tokens. SE recalibrates the token feature channels, and the Transformer models long-range interactions. A residual bridge combines the Transformer output with the original convolutional tokens. The fused sequence is then passed to the BiLSTM for ordered bidirectional refinement.

### 3.2. Convolutional Tokenisation

**Figure 2:**
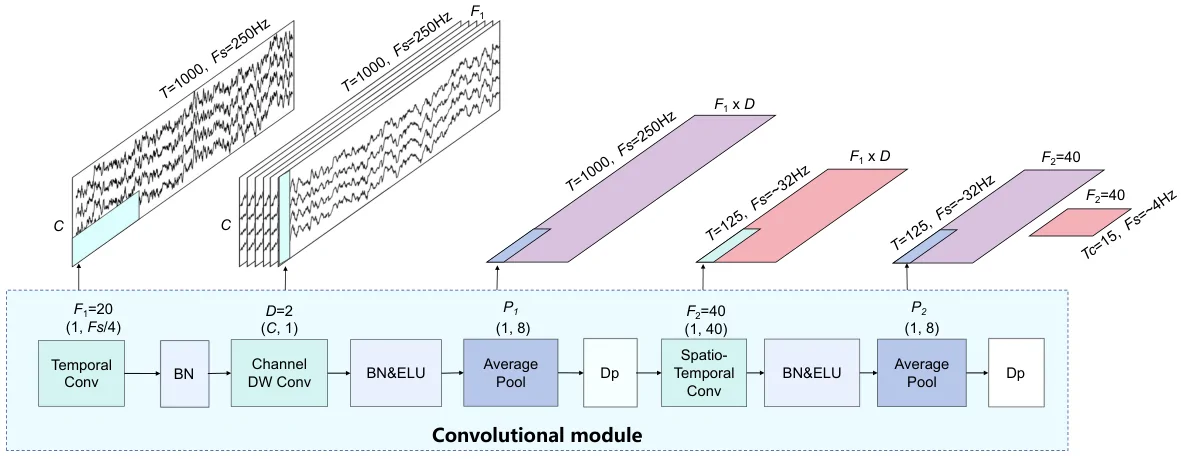
Structure of the convolutional tokenisation module.

The convolutional front-end, whose architecture is depicted in Figure 2, converts raw EEG into compact temporal tokens while retaining the underlying local structure. It contains temporal convolution, depthwise spatial convolution, and spatio-temporal convolution. The temporal stage uses 20 kernels of size 1×64 as a learnable filter bank. The depthwise convolution spans all *C* electrodes with a depth multiplier of two and expands the feature dimension to 40. A subsequent 1×16 convolution aggregates local spatio-temporal context. Batch normalisation follows each convolution, while ELU activations, two 1×8 average-pooling operations, and dropout regularise and compress the representation.

The final feature map is rearranged into a sequence

(1) $$F=\phi \left(X;\theta_{c}\right)\in R^{B\times T_{c}\times d},$$

where d=40 and

(2) $$T_{c}=\left\lfloor \frac{\left\lfloor T/P_{1}\right\rfloor }{P_{2}}\right\rfloor ,\;P_{1}=P_{2}=8.$$

For the 1000-sample BCI inputs, $T_{c}=15$. The local representation F is retained for the residual bridge.

### 3.3. Feature-Channel Recalibration

**Figure 3:**
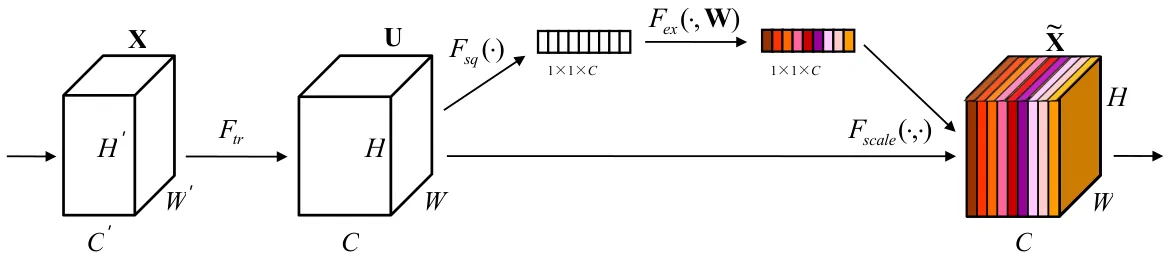
Principle of SE-based feature-channel recalibration.

The SE block is placed before the Transformer to model dependencies among the learned convolutional feature channels [37]. Its principle is illustrated in Figure 3. Given F, temporal averaging forms a descriptor for each feature channel:

(3) $$z_{c}=\frac{1}{T_{c}}\sum_{t=1}^{T_{c}}F_{t,c}.$$

Two fully connected layers produce channel weights that rescale the token sequence

(4) $$F_{\mathrm{SE}}=F⊙\sigma \;\left(W_{2}\delta \left(W_{1}z\right)\right),$$

where δ(·) and σ(·) denote ReLU and sigmoid functions. SE recalibrates learned feature channels rather than the original EEG electrodes.

### 3.4. Global Context and Residual Fusion

A learnable positional embedding is added to $F_{\mathrm{SE}}$. Three Transformer layers with four attention heads model trial-wide dependencies. For head *i*,

(5) $$A_{i}=\mathrm{softmax}\;\left(\frac{Q_{i}K_{i}^{T}}{\sqrt{d_{k}}}\right)V_{i}.$$

The head outputs are concatenated and projected. Each attention or feed-forward sublayer is followed by dropout, residual addition, and layer normalisation; the feed-forward network uses GELU activation.

Let G denote the Transformer output. The cross-stage bridge is

(6) H=G+F.

The bridge supplies the BiLSTM with the contextualised representation and a direct path to the original convolutional tokens. No residual connection is applied around the BiLSTM.

### 3.5. Bidirectional Sequential Refinement

**Figure 4:**
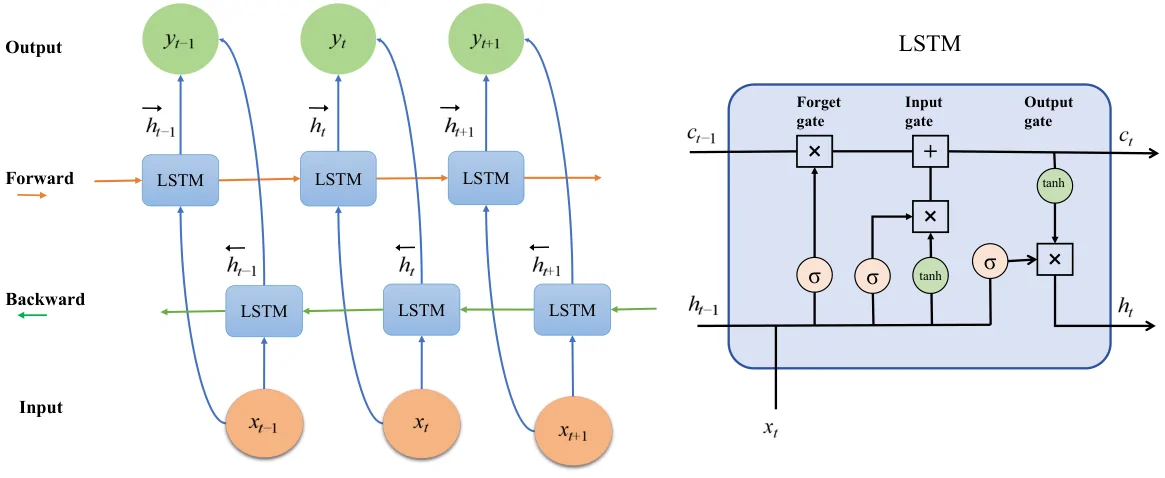
Principle of bidirectional LSTM refinement.

Self-attention establishes relationships between all compressed tokens but does not explicitly propagate a gated state in forward and backward time. The BiLSTM therefore operates on the fused sequence H, as illustrated in Figure 4. For the forward direction, the gates are

(7) $$g_{t}=\sigma \;\left(W_{g}\left[H_{t};h_{t-1}\right]+b_{g}\right),\;g\in \left\{i,f,o\right\},$$

with separate parameters in the backward direction. The two hidden states are concatenated:

(8) $$h_{t}=\left[{\vec{h}}_{t};{\overset{\leftarrow }{h}}_{t}\right].$$

The final forward and backward states form an 80-dimensional trial-level representation for the dropout-regularised classifier.

### 3.6. Datasets and Preprocessing

BCI Competition IV-2a contains four-class MI recordings from nine subjects (A01–A09) using 22 EEG channels at 250 Hz. The classes are left hand, right hand, both feet, and tongue imagery. A 4 s segment from 2 to 6 s after cue onset produced a 22×1000 input [38].

BCI Competition IV-2b contains nine right-handed subjects performing left- and right-hand MI, recorded from C3, Cz, and C4 at 250 Hz. Sessions 1–3 provide development data and sessions 4–5 provide evaluation data. A 4 s segment from 3 to 7 s produced a 3×1000 input [38].

The PhysioNet EEG Motor Movement/Imagery database contains 64-channel recordings from 109 subjects, acquired with BCI2000 at 160 Hz using the international 10–10 montage [39]. Four imagery tasks were retained: left fist, right fist, both fists, and both feet. Following Zhou et al. [27], Subjects 38, 88, 89, 92, 100, and 104 were excluded, leaving 103 subjects. Four-second segments produced 64×640 inputs.

**Figure 5:**
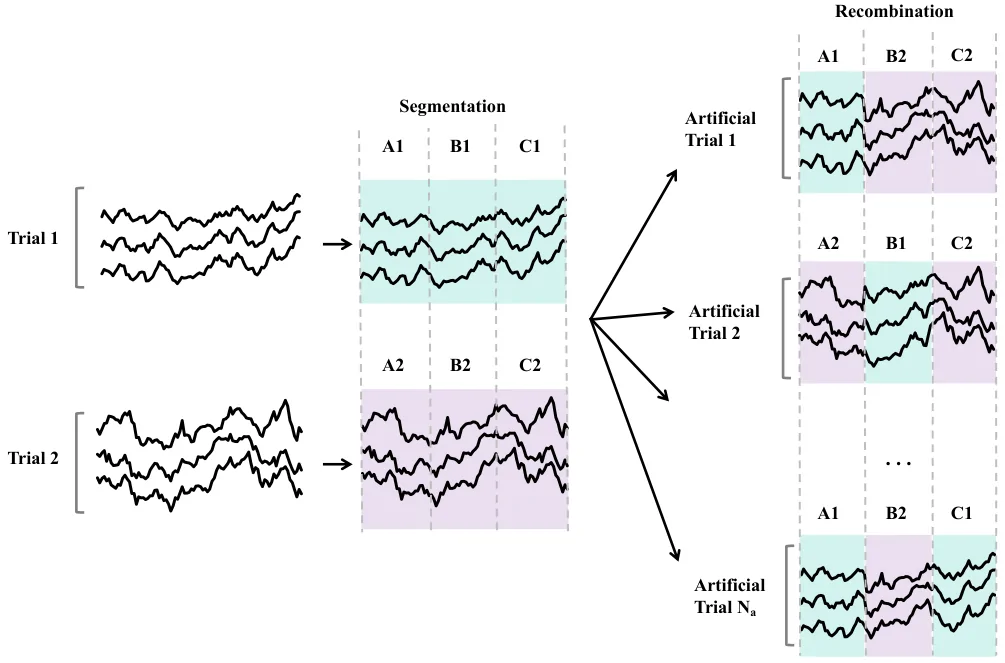
Principle of segmentation-and-recombination data augmentation.

Z-score normalisation used the mean and standard deviation estimated from the training data and applied them unchanged to validation and test trials. No additional filtering or artefact removal was introduced beyond the preprocessing supplied with the public datasets. Training data were augmented by segmentation and recombination (S&R) [40], as illustrated in Figure 5. Each trial was divided into eight temporally aligned segments. For each temporal position, a segment was sampled from a randomly selected training trial of the same class and placed in the corresponding position of the synthetic trial. This preserves coarse temporal alignment and class membership while introducing within-class variation. It can nevertheless interrupt continuity across segment boundaries, which motivated the augmentation ablation reported in Section 4.1. No test trials were used to construct the synthetic samples.

### 3.7. Experimental Configuration

Subject-dependent evaluation followed the official splits for BCI IV-2a (session 1 for development and session 2 for testing) and IV-2b (sessions 1–3 for development and sessions 4–5 for testing), with 30% of each development set reserved for validation. The BCI protocol was implemented independently for each subject. Candidate configurations were trained and validated without access to the locked test partitions. For PhysioNet, pooled trials were stratified into a 10% locked test set, after which 20% of the remaining trials were assigned to validation and the rest to training. Because trials from the same subject can occur in different partitions, this pooled protocol is a mixed-subject evaluation rather than an unseen-subject test.

Models were implemented in PyTorch 2.8.0 and trained on an NVIDIA GeForce RTX 4090 GPU. Adam was used with an initial learning rate of 0.001, $\beta_{1}=0.5$, and $\beta_{2}=0.999$. Mini-batches of 288 were optimised with cross-entropy loss. Following the fixed training schedules used in the reproduced MSCFormer and CLTNet implementations, every model received the same 1000-epoch training budget. Early stopping was not used; instead, the checkpoint with the lowest validation loss over the full schedule was selected before evaluation on the locked test set. This protocol provides all architectures with the same maximum training budget and applies a consistent validation-based checkpoint-selection criterion.

Each experiment was repeated with random seeds for 2025, 2026, and 2027. For the two BCI datasets, the three runs were first summarised for each subject, and inferential tests used the nine subject-level means. Accuracy and Cohen’s kappa were reported, with overall BCI accuracy expressed as mean ± SD across participants. Paired differences between STDR-Net and each comparator were evaluated with exact two-sided Wilcoxon signed-rank tests. We report rank-biserial correlations and percentile-bootstrap 95% confidence intervals for the mean paired accuracy difference. Holm correction controlled the family-wise error rate separately for the nine baseline comparisons and six ablation comparisons within each BCI dataset. For PhysioNet, trials from all participants were pooled before stratified splitting, and performance is reported descriptively across three runs. Because subjects may occur in multiple splits, these results represent pooled trial-level performance rather than generalisation to unseen subjects.

Computational profiles were measured on the same RTX 4090. Parameter counts include trainable parameters, and FLOPs refer to one BCI IV-2a trial. Inference latency was averaged over 100 repetitions.

## 4. Results

### 4.1. Ablation Study

Six variants removed the Transformer, BiLSTM, SE block, residual bridge, or S&R augmentation, or reversed the two global and sequential stages to form BiLSTM→Transformer. Table 2 and Table 3 summarise mean accuracy across subjects and subject-level paired comparisons.

On IV-2a, every ablation reduced mean accuracy, with decreases from 1.49 percentage points for removal of the bridge to 8.28 points for removal of the BiLSTM. All six paired comparisons remained significant after Holm correction. The bridge-specific and ordering results directly support the proposed information path on this dataset. S&R augmentation also produced a sizeable improvement, indicating that its empirical benefit outweighed the possible discontinuity introduced at segment boundaries.

The IV-2b results were less conclusive. Accuracy differences were within 0.81 percentage points, the bridge-free variant was 0.13 points higher than the full model, and no comparison remained significant after correction. The ablations therefore show dataset-dependent evidence rather than a uniform advantage for every component.

### 4.2. Hyperparameter Sensitivity

**Figure 6:**
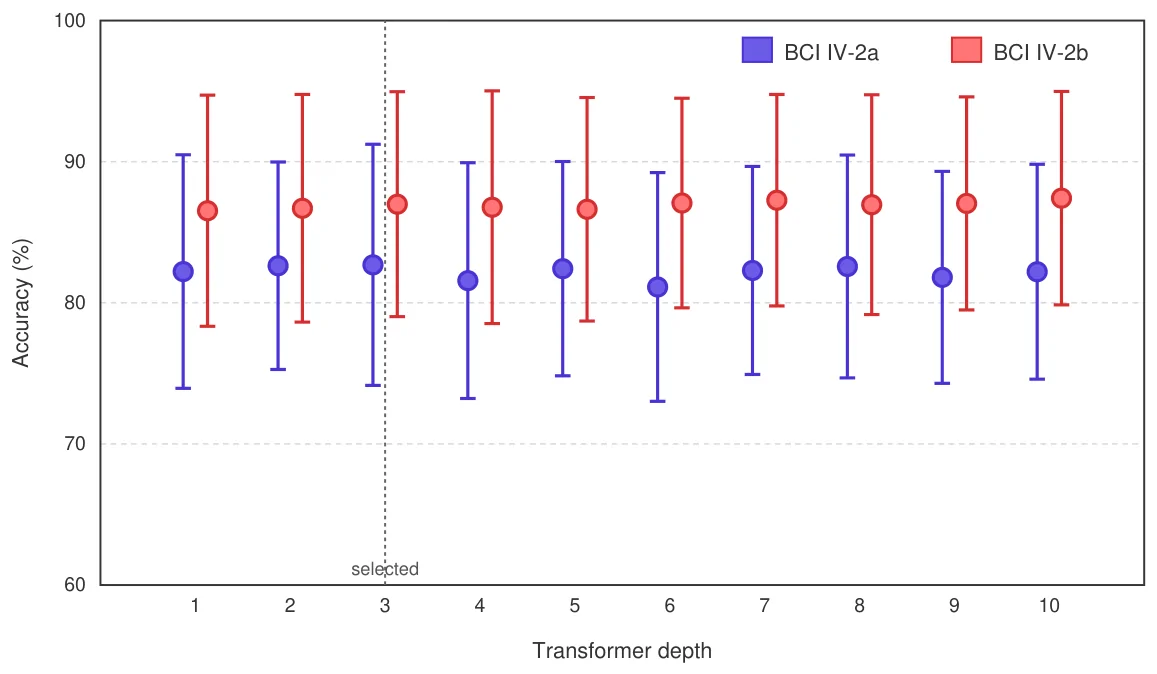
Sensitivity of recognition accuracy to Transformer depth on BCI IV-2a and IV-2b. Points show subject means and error bars show 95% confidence intervals. The dashed line marks the selected depth.

Transformer depth is an important hyperparameter that influences model capacity and recognition performance. The recognition accuracies of BCI IV-2a and IV-2b at different Transformer depths are shown in Figure 6. IV-2a was more sensitive to depth: mean accuracy ranged from 81.12% to 82.69%, with the highest value at three layers. IV-2b varied by less than one percentage point across depths 1–10, indicating low sensitivity to this parameter. Three layers were retained because they gave the best IV-2a result and remained within the stable IV-2b range.

**Figure 7:**
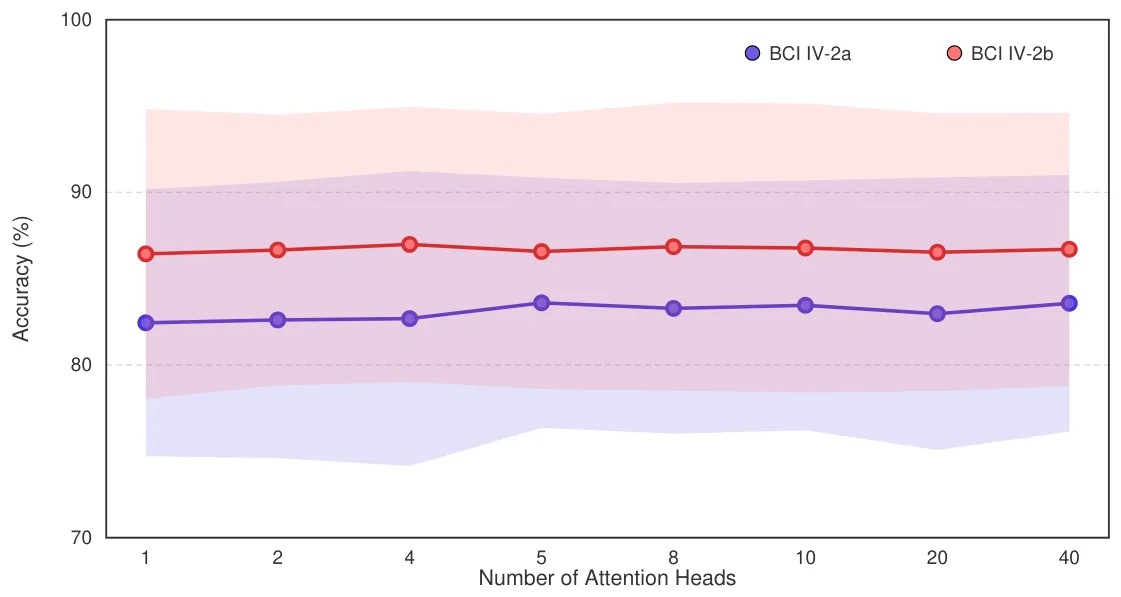
Sensitivity of recognition accuracy to the number of attention heads on BCI IV-2a and IV-2b. The shaded areas indicate 95% confidence intervals.

The number of attention heads is another important hyperparameter. The results for different numbers of attention heads are shown in Figure 7. On IV-2a, five heads gave the highest mean accuracy of 83.60%. On IV-2b, four heads gave the highest mean accuracy of 86.99%. Four heads were retained as the common configuration because this setting was optimal on IV-2b and remained close to the IV-2a maximum while providing an equal 10-dimensional subspace per head for the 40-dimensional tokens.

### 4.3. Comparison with Baselines

Nine representative models were reproduced: ShallowConvNet [7], DeepConvNet [7], EEGNet [8], MBEEGNet [17], EEGTCNet [11], Conformer [12], MSCFormer [13], ADFCNN [9], and CLTNet [14]. Baseline architectures followed their publications, while dataset partitions, augmentation, optimisation, and evaluation procedures were aligned as closely as their input requirements allowed. Table 4 and Table 5 report the results on BCI IV-2a and IV-2b, respectively.

**Figure 8:**
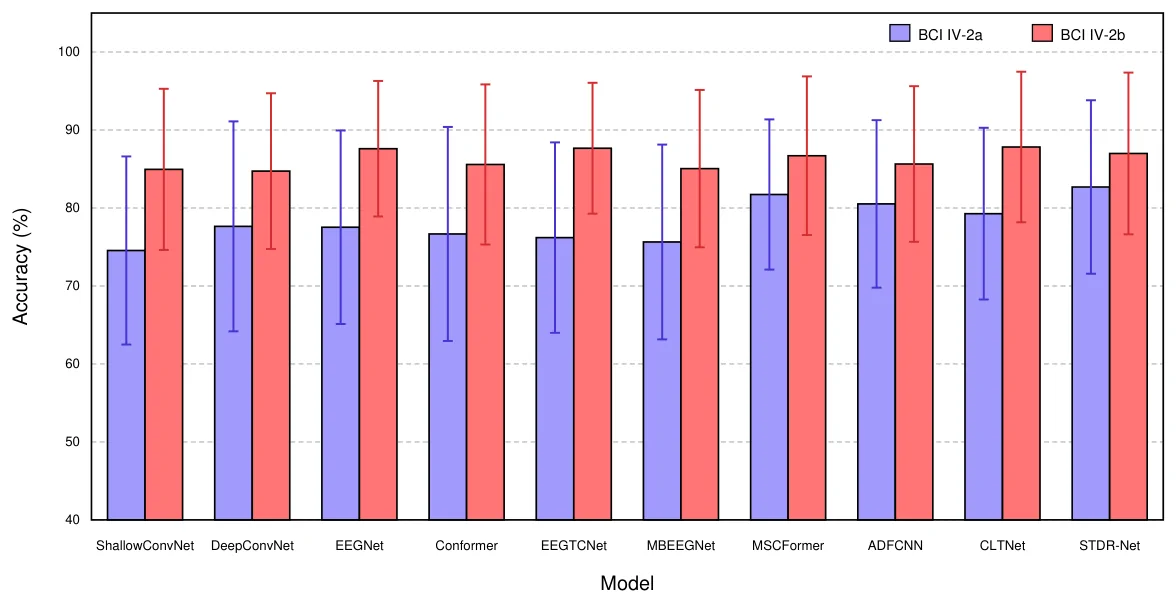
Recognition accuracy of STDR-Net and nine reproduced baselines on BCI IV-2a and IV-2b under the same subject-dependent evaluation protocol.

STDR-Net obtained the highest observed mean accuracy on IV-2a. Its 0.96-point difference from MSCFormer had a confidence interval that crossed zero and was not significant after correction. Significant corrected differences remained for ShallowConvNet, DeepConvNet, MBEEGNet, and Conformer. On IV-2b, CLTNet, EEGTCNet, and EEGNet had slightly higher mean accuracies than STDR-Net, and none of the nine paired comparisons were significant after correction. Figure 8 visualises the corresponding mean accuracies. These results distinguish numerical ranking from statistically supported differences.

Table 6 shows that, on PhysioNet, ADFCNN had the highest observed mean accuracy (60.43%), followed by STDR-Net (59.76%) and DeepConvNet (59.54%). These are descriptive pooled-split results and do not establish cross-subject generalisation.

### 4.4. Computational Cost

Table 7 compares model size and measured computational cost. STDR-Net used 0.118 million parameters and 0.071 GFLOPs per trial. Its parameter count was close to CLTNet and below DeepConvNet and Conformer. Its FLOPs were below ShallowConvNet, Conformer, and MSCFormer, but its measured latency was higher than that of several compact convolutional models. STDR-Net therefore offers moderate computational cost rather than minimum complexity.

### 4.5. Visualisation

**Figure 9:**
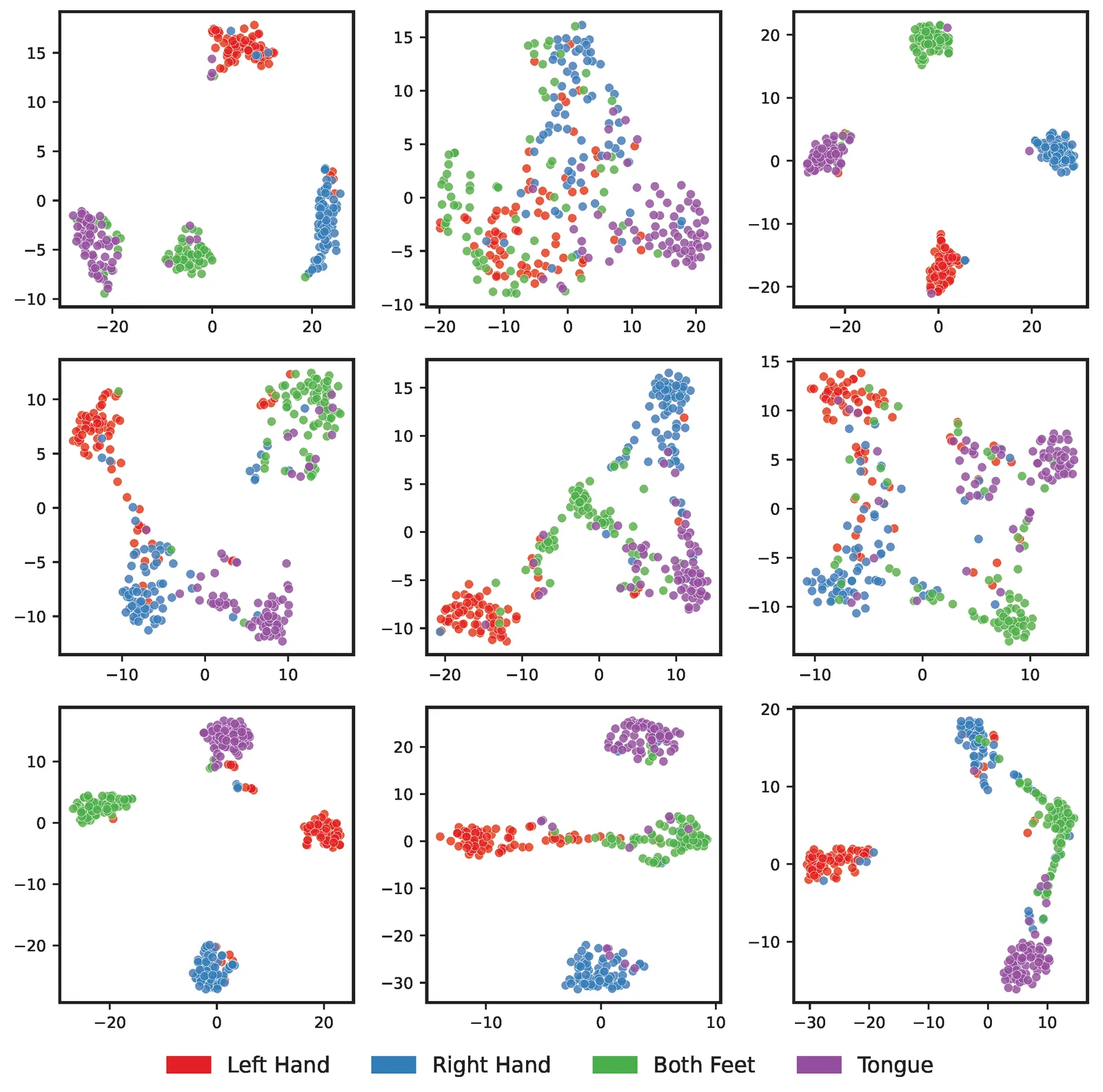
t-SNE projections of the 80-dimensional pre-classification features for the nine BCI IV-2a subjects. Each point represents an EEG trial, and colours indicate the MI classes.

To inspect the class structure of the learned representation, t-distributed stochastic neighbour embedding (t-SNE) was applied to the 80-dimensional feature vector produced by the BiLSTM. Figure 9 shows two-dimensional projections for the nine IV-2a subjects. A03 and A07 exhibit comparatively compact and separated clusters, whereas A02 and A06 show greater overlap. Because t-SNE is stochastic and can distort global geometry, these plots are treated as qualitative views rather than quantitative evidence of separability.

**Figure 10:**
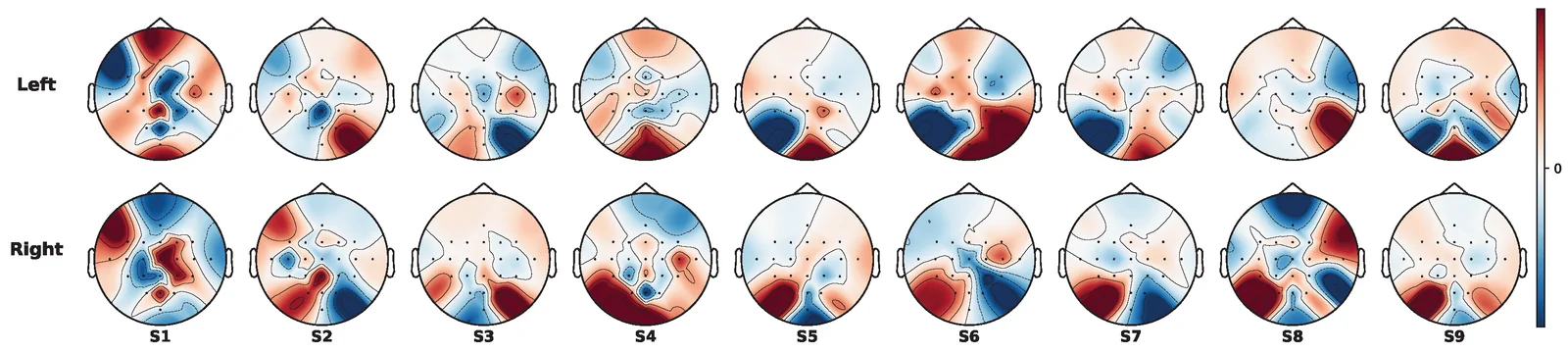
Exploratory class activation topographies for left- and right-hand imagery across the nine BCI IV-2a subjects. Red and blue indicate positive and negative normalised CAT values, respectively; stronger colour intensity indicates a larger magnitude.

Class activation topography (CAT) was computed following the Conformer visualisation framework [12]. Input × Gradient saliency maps estimated the sensitivity of class scores to the EEG input. Trial-averaged EEG and saliency maps were separately z-score-normalised, multiplied element-wise, averaged over time, and projected onto the 22-channel montage. Figure 10 shows subject- and class-dependent patterns. Some subjects exhibit lateralised central or centro-parietal sensitivity, while others show distributed patterns. These maps are exploratory model-attribution results. Input × Gradient attributions are model- and input-dependent and may be sensitive to small input perturbations. Accordingly, the apparent lateralised patterns in these qualitative maps should be interpreted as exploratory model-attribution results rather than evidence of cortical activation or physiological validity.

## 5. Discussion

To examine the proposed design, we conducted ablation experiments. On IV-2a, removing the residual bridge reduced mean accuracy by 1.49 percentage points, while placing the BiLSTM before the Transformer reduced it by 2.07 points. Both differences remained significant after Holm correction. These findings support the hypothesis that the recurrent stage benefits from receiving a fused local–global representation. Taken together, the larger performance reductions associated with removing the Transformer, BiLSTM, SE block, or augmentation indicate that the IV-2a performance of the full model reflects the combined contribution of multiple components, rather than the residual bridge alone. Nevertheless, the ablation analysis is not exhaustive. Further comparisons with alternative fusion strategies, such as feature concatenation or using the Transformer output alone, would help disentangle the contributions of the individual information pathways and represent an important direction for future work.

The IV-2b evidence is substantially weaker. All ablation differences were below one percentage point, and none remained significant after correction. The full model was also not the numerical leader among the reproduced IV-2b baselines. The binary task has only three EEG channels, and several compact models already performed strongly. The benefit of the dependency-refinement path should therefore be interpreted as task-dependent rather than universal.

The baseline comparisons lead to the same distinction between observed means and statistical superiority. STDR-Net had the highest IV-2a mean, but its differences from MSCFormer, ADFCNN, CLTNet, EEGNet, and EEGTCNet were not significant after Holm correction. On IV-2b, three baselines had slightly higher observed means and no corrected comparison was significant. With nine participants, the Wilcoxon analysis has limited power; the paired confidence intervals and effect sizes therefore provide important context for small differences.

On PhysioNet, STDR-Net was competitive but did not have the highest mean accuracy. More importantly, the pooled trial-level split permits trials from the same subject to appear in training and test partitions. It can therefore reflect subject-specific characteristics and cannot support a claim of generalisation to unseen subjects. A subject-wise split or leave-one-subject-out protocol is needed to evaluate cross-subject transfer.

The computational analysis shows that the performance cannot be considered independently of model cost. STDR-Net has a moderate parameter count and FLOP requirement, but it is not the smallest or fastest model. Its value lies in the accuracy–cost balance under the present hardware and protocol, not in computational minimality. Latency and training time will also depend on implementation, input length, batch size, and device.

The S&R ablation supports augmentation on IV-2a but not IV-2b. Sampling temporally aligned segments from same-class trials preserves segment order and coarse alignment, but concatenation can disrupt dependencies across segment boundaries. Future work could compare S&R with continuity-preserving augmentation and quantify whether the Transformer and BiLSTM rely on boundary artefacts.

Finally, the t-SNE and CAT figures aid qualitative inspection but do not prove physiological validity. Future work should quantify hemispheric lateralisation, assess attribution stability, and compare the maps with independently defined sensorimotor regions. Broader validation should include unseen-subject protocols, external datasets, and larger subject samples.

## 6. Conclusions

STDR-Net combines convolutional tokenisation, feature-channel recalibration, global self-attention, a cross-stage residual bridge, and bidirectional temporal refinement. It achieved the highest observed mean accuracy on BCI IV-2a and competitive performance on BCI IV-2b and PhysioNet. Bridge-specific and ordering ablations supported the proposed information path on IV-2a, but not on IV-2b, where corrected differences were not significant. The results therefore support dependency refinement as a useful, dataset-dependent design rather than establishing uniform superiority. Evaluation on completely unseen subjects remains necessary.

## Figures and Tables

**Table 1:** Layer-wise configuration of STDR-Net.

Stage	Configuration	Output Shape
Input	Raw trial	(B,1,C,T)
Temporal convolution	20 filters, 1×64	(B,20,C,T)
Spatial convolution	Depthwise, D=2	(B,40,1,T)
Pooling 1	Average, 1×8	(B,40,1,⌊T/8⌋)
Spatio-temporal convolution	40 filters, 1×16	(B,40,1,⌊T/8⌋)
Pooling 2 and tokenisation	Average, 1×8	$\left(B,T_{c},40\right)$
SE recalibration	Reduction ratio r=4	$\left(B,T_{c},40\right)$
Transformer encoder	3 layers, 4 heads	$\left(B,T_{c},40\right)$
Residual bridge	Transformer output + CNN tokens	$\left(B,T_{c},40\right)$
BiLSTM	40 units per direction	$\left(B,T_{c},80\right)$
Classifier	Dropout and dense layer	$\left(B,N_{\mathrm{cls}}\right)$

**Table 2:** Ablation results on BCI Competition IV-2a.

Variant	Accuracy (%)	Δ (pp), 95% CI	$r_{\mathrm{rb}}$	$p_{\mathrm{Holm}}$
w/o Transformer	77.15±14.72	5.54 [3.16, 8.28]	0.956	0.0391 *
w/o BiLSTM	74.41±16.90	8.28 [3.90, 13.01]	0.956	0.0391 *
w/o SE	77.71±15.22	4.98 [2.11, 7.97]	0.911	0.0391 *
w/o residual bridge	81.20±11.33	1.49 [0.40, 2.65]	0.756	0.0429 *
w/o augmentation	76.26±9.25	6.43 [4.46, 8.36]	1.000	0.0234 *
BiLSTM→Transformer	80.62±11.76	2.07 [0.82, 3.40]	0.867	0.0391 *
STDR-Net (full)	82.69±11.12	–	–	–

Accuracy is mean ± SD across subjects (n=9). Δ is STDR-Net minus the variant. $r_{rb}$ is the rank-biserial correlation. * $p_{\mathrm{Holm}}<0.05$. Bold indicates the highest observed value in the corresponding column.

**Table 3:** Ablation results on BCI Competition IV-2b.

Variant	Accuracy (%)	Δ (pp), 95% CI	$r_{\mathrm{rb}}$	$p_{\mathrm{Holm}}$
w/o Transformer	86.36±10.45	0.63 [−0.25, 1.49]	0.489	0.9375
w/o BiLSTM	86.20±10.46	0.79 [0.20, 1.40]	0.622	0.6328
w/o SE	86.18±10.27	0.81 [−0.07, 1.53]	0.511	0.9375
w/o residual bridge	87.12±9.82	−0.13 [−0.80, 0.60]	−0.133	1.0000
w/o augmentation	86.42±8.75	0.57 [−0.85, 2.04]	0.311	1.0000
BiLSTM→Transformer	86.42±10.14	0.57 [−0.30, 1.52]	0.289	1.0000
STDR-Net (full)	86.99±10.37	–	–	–

Accuracy is mean ± SD across subjects (n=9). Δ is STDR-Net minus the variant. No comparison remained significant after Holm correction. Bold indicates the highest observed value in the corresponding column.

**Table 4:** Comparison with reproduced baselines on BCI Competition IV-2a.

Method	Accuracy (%)	Cohen’s κ	Δ (pp), 95% CI	$r_{\mathrm{rb}}$	$p_{\mathrm{Holm}}$
ShallowConvNet	74.55±12.06	0.6607	8.14 [5.36, 11.10]	1.000	0.0352 *
DeepConvNet	77.64±13.46	0.7018	5.05 [3.43, 7.06]	1.000	0.0352 *
EEGNet	77.53±12.41	0.7004	5.16 [1.14, 9.27]	0.733	0.2188
MBEEGNet	75.64±12.49	0.6752	7.05 [5.52, 8.67]	1.000	0.0352 *
EEGTCNet	76.20±12.21	0.6826	6.49 [3.03, 10.04]	0.911	0.0586
Conformer	76.67±13.72	0.6890	6.02 [4.22, 8.04]	1.000	0.0352 *
ADFCNN	80.52±10.74	0.7402	2.17 [−0.13, 4.96]	0.556	0.3125
CLTNet	79.27±11.01	0.7236	3.42 [0.26, 6.85]	0.689	0.2227
MSCFormer	81.73±9.63	0.7563	0.96 [−1.38, 3.19]	0.333	0.4258
STDR-Net (proposed)	82.69±11.12	**0.7692**	–	–	–

Δ is STDR-Net minus the comparator. Exact two-sided Wilcoxon tests were Holm-corrected across nine comparisons. * $p_{\mathrm{Holm}}<0.05$. Bold indicates the highest observed value in the corresponding column.

**Table 5:** Comparison with reproduced baselines on BCI Competition IV-2b.

Method	Accuracy (%)	Cohen’s κ	Δ (pp), 95% CI	$r_{\mathrm{rb}}$	$p_{\mathrm{Holm}}$
ShallowConvNet	84.95±10.33	0.6990	2.04 [1.12, 2.79]	0.956	0.0703
DeepConvNet	84.73±9.98	0.6946	2.26 [0.96, 3.91]	0.956	0.0703
EEGNet	87.60±8.69	0.7520	−0.61 [−2.24, 1.00]	−0.333	1.0000
MBEEGNet	85.05±10.09	0.7010	1.94 [0.65, 3.03]	0.844	0.1641
EEGTCNet	87.66±8.39	0.7533	−0.67 [−2.35, 0.93]	−0.156	1.0000
Conformer	85.58±10.27	0.7115	1.41 [0.03, 2.77]	0.644	0.4883
ADFCNN	85.64±9.98	0.7129	1.34 [0.46, 2.22]	0.889	0.1641
CLTNet	87.82±9.65	**0.7564**	−0.83 [−1.70, 0.17]	−0.600	0.4883
MSCFormer	86.70±10.17	0.7340	0.29 [−0.67, 1.19]	0.156	1.0000
STDR-Net (proposed)	86.99±10.37	0.7398	–	–	–

Δ is STDR-Net minus the comparator. No comparison remained significant after Holm correction. Bold indicates the highest observed value in the corresponding column.

**Table 6:** Comparison on the PhysioNet EEG Motor Movement/Imagery dataset.

Method	Accuracy (%)	Cohen’s κ
ShallowConvNet	59.48±0.23	0.46
DeepConvNet	59.54±0.94	0.46
EEGNet	53.20±0.31	0.38
MBEEGNet	57.54±1.77	0.43
EEGTCNet	59.10±1.90	0.45
Conformer	56.95±1.32	0.43
ADFCNN	60.43±0.86	**0.47**
CLTNet	56.09±0.81	0.41
MSCFormer	58.41±0.98	0.45
STDR-Net (proposed)	59.76±1.44	0.46

Accuracy is mean ± SD across independent runs; kappa is the corresponding mean. Bold indicates the highest observed value in the corresponding column.

**Table 7:** Computational costs on BCI Competition IV-2a.

Method	Parameters (M)	FLOPs/Trial (G)	Latency/Trial (ms)	Training Time/Epoch (s)
ShallowConvNet	0.046	0.112	0.135	0.079
DeepConvNet	0.283	0.066	0.417	0.034
EEGNet	0.003	0.046	0.404	0.023
MBEEGNet	0.007	0.067	1.030	0.043
EEGTCNet	0.005	0.014	0.826	0.021
Conformer	0.166	0.126	2.199	0.053
ADFCNN	0.005	0.002	0.854	0.060
CLTNet	0.120	0.071	1.473	0.035
MSCFormer	0.094	0.143	1.467	0.052
STDR-Net (proposed)	0.118	0.071	1.615	0.046

## Data Availability

The BCI Competition IV-2a and IV-2b datasets are publicly available from the BCI Competition IV repository at https://www.bbci.de/competition/iv/ (accessed on 10 January 2025). The PhysioNet EEG Motor Movement/Imagery database is publicly available at https://doi.org/10.13026/C28G6P. No new datasets were created for this study.
